# The *Brassica napus* seed microbiota is cultivar‐specific and transmitted via paternal breeding lines

**DOI:** 10.1111/1751-7915.14077

**Published:** 2022-05-20

**Authors:** Birgit Wassermann, Ahmed Abdelfattah, Wisnu Adi Wicaksono, Peter Kusstatscher, Henry Müller, Tomislav Cernava, Simon Goertz, Steffen Rietz, Amine Abbadi, Gabriele Berg

**Affiliations:** ^1^ ACIB GmbH Petersgasse 14 8010 Graz Austria; ^2^ Institute of Environmental Biotechnology Graz University of Technology Petersgasse 12 Graz 8010 Austria; ^3^ Leibniz Institute for Agricultural Engineering and Bioeconomy (ATB) Max‐Eyth Allee 100 14469 Potsdam Germany; ^4^ NPZ Innovation GmbH Hohenlieth‐Hof 24363 Holtsee Germany; ^5^ Institute for Biochemistry and Biology University of Potsdam 14476 Potsdam OT Golm Germany

## Abstract

Seed microbiota influence germination and plant health and have the potential to improve crop performance, but the factors that determine their structure and functions are still not fully understood. Here, we analysed the impact of plant‐related and external factors on seed endophyte communities of 10 different oilseed rape (*Brassica napus* L.) cultivars from 26 field sites across Europe. All seed lots harboured a high abundance and diversity of endophytes, which were dominated by six genera: *Ralstonia*, *Serratia*, *Enterobacter*, *Pseudomonas*, *Pantoea*, and *Sphingomonas*. The cultivar was the main factor explaining the variations in bacterial diversity, abundance and composition. In addition, the latter was significantly influenced by diverse biotic and abiotic factors, for example host germination rates and disease resistance against *Plasmodiophora brassicae*. A set of bacterial biomarkers was identified to discriminate between characteristics of the seeds, for example *Sphingomonas* for improved germination and *Brevundimonas* for disease resistance. Application of a Bayesian community approach suggested vertical transmission of seed endophytes, where the paternal parent plays a major role and might even determine the germination performance of the offspring. This study contributes to the understanding of seed microbiome assembly and underlines the potential of the microbiome to be implemented in crop breeding and biocontrol programmes.

## Graphical abstract

Seed microbiota influence germination and plant health and have the potential to improve crop performance. Analyzing the impact of plant‐related traits and external factors on the seed microbiomes of ten oilseed rape cultivars from 26 fields across Europe, revealed the cultivar as the main driver of bacterial diversity, abundance, and composition. We identified bacterial biomarkers for pathogen resistance and germination rates and observed that the paternal parent plays a role during the vertical transmission of seed endophytes.

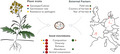

## Introduction

The inner and outer tissues of plants are colonized by diverse and abundant microbial communities, respectively, referred to as endophytes and epiphytes (Berg *et al*., 2005; Hardoim *et al*., 2015). They can form complex interactions with their host plant and the observed stability of the overall system suggests evolutionary selection between plants and microorganisms, eponymous for the hologenome concept of evolution (Zilber‐Rosenberg and Rosenberg, 2008). In support of this, phylosymbiosis, describing the tendency of closer related plant species to host similar microbial communities (Brucker and Bordenstein, 2013; Theis *et al*., 2016), was found to be stronger for endophytes than for epiphytes (Mazel *et al*., 2018; Mendes *et al*., 2018; Kim *et al*., 2020). Endophytes interact intimately with their host and take advantage of nutrient availability and shelter from unfavourable external conditions, exhibiting a range of plant beneficial traits in return (Hardoim *et al*., 2015; Berg *et al*., 2020). Most recently, particular benefits for the host plant have been proposed for microbiota found inside seeds (Berg and Raaijmakers, 2018; Simonin *et al*., 2022). As previously suggested for plants in general (Truyens *et al*., 2015; Shade *et al*., 2017) and recently proven for tomato (Bergna *et al*., 2018), oak (Abdelfattah *et al*., 2021b) and wheat (Walsh *et al*., 2021), a specific fraction of seed‐associated microbiota is constantly and continuously transmitted across plant generations to the developing seedling's phyllosphere and rhizosphere. Interestingly, this effect was even more pronounced for native plants (Wassermann *et al*., 2019). The establishment of such an intimate relationship suggests that seed‐associated microbiota fulfil important biological and ecological functions for their host, for example by exerting a priority effect on subsequent colonizers once the seedling germinates (Abdelfattah *et al*., 2021b). Seed microorganisms were shown to produce phytohormones and antimicrobial compounds, fix and mobilize nutrients from the environment, defend against disease and manipulate host genes and hormones for increased resilience towards environmental stresses (Berg and Raaijmakers, 2018; Bergna *et al*., 2018; Dai *et al*., 2020; Matsumoto *et al*., 2021). Harnessing the ‘phytomicrobiome’ is, thus, proposed as the most promising approach to improve farm productivity while safeguarding environmental health (Singh and Trivedi, 2017).

*Brassica napus* L. (oilseed rape) is among the most important crops worldwide, with more than 86 million tons produced each year (FAO, 2020). However, production systems are substantially affected by a steadily increasing incidence of field diseases, often caused by soil‐borne pathogens such as *Verticillium longisporum* or *Plasmodiophora brassicae* (Bennett *et al*., 2012; Rybakova *et al*., 2020), which are particularly difficult to suppress (Bakker *et al*., 2020). Here, plant microbiome‐based strategies represent promising solutions (Rybakova *et al*., 2017; Zhao *et al*., 2017; Jiménez‐Gómez *et al*., 2020). Recently, a number of studies targeted to decipher the microbiome of oilseed rape seeds, and several biotic and abiotic factors have been described to contribute to its assembly: while seed epiphytes were found to be mainly influenced by environmental factors at a certain field site (Morales Moreira *et al*., 2021), the host genotype played a significant role when epi‐ and endophytes were analysed as a collective (Rochefort *et al*., 2019). In addition, the seed microbiome has been suggested to contribute to pathogen resistance, where low bacterial diversity correlated positively with higher counts of potential pathogens and with a greater accessibility to pathogenic as well as beneficial microbial inoculants (Rybakova *et al*., 2017). However, research is still needed to understand the seed microbiome assembly and transmission.

In the present study, we analysed 16S rRNA gene profiles of seed endophytes of 10 different oilseed rape cultivars, harvested from 26 different fields in five European countries, to answer the following questions: (i) how important is the impact of the cultivar and the field site on seed endophyte composition?; (ii) are functional characteristics of the host, such as germination rate and resistance to pathogens, associated with distinct seed endophytes?; and if so, (iii) could a beneficial microbiota, that is vertically transmitted, assist the next plant generation to cope with the environmental conditions at distinct field sites?; and finally (iv) can we quantify the contribution of maternal and paternal breeding lines to the seed microbiome of a cross‐fertilized hybrid offspring? By answering these questions, we provide deepening insights into the seed microbiome of oilseed rape with potential implications for seed production, breeding strategies and the development of targeted bacterial seed treatments.

## Results

### The general structure of the oilseed rape seed microbiota

After demultiplexing raw reads and joining forward and reversed reads, 603 275 mean high‐quality reads per sample were recovered. Taxonomic assignment, however, found a high number of non‐target taxa (chloroplasts, mitochondria, etc.), which were removed, resulting in 233 025 mean high‐quality reads per sample and a total of 23 543 ASVs. After normalization to 10 000 reads per samples, a mean value of 265 bacterial ASVs per sample remained. Overall, *Gammaproteobacteria* was the dominating class (77.8%), followed by *Alphaproteobacteria* (8.8%), *Bacteroida* (8.0%) and *Bacilli* and *Actinobacteria* (each 2.2%).

### Host‐related and external factors influencing the oilseed rape seed microbiota

**Fig. 1:**
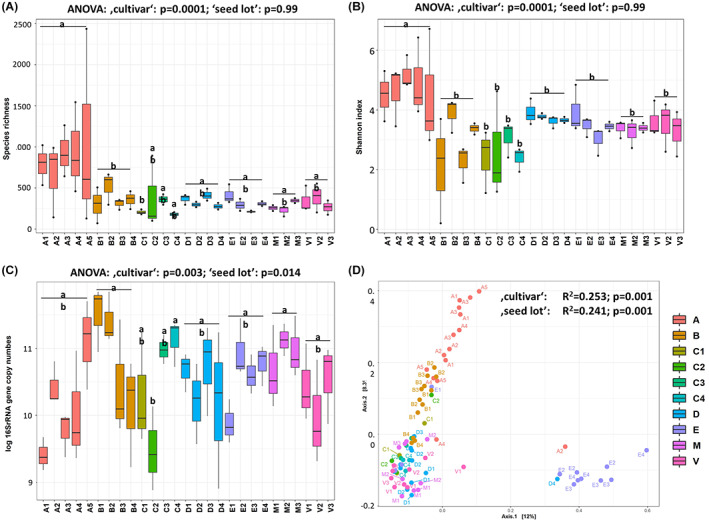
Box plots showing bacterial species richness (A), Shannon diversity (B) and abundance (C) in seeds. Samples are represented as seed lots and colour‐grouped by the respective cultivar. Results of statistical significance are reported on top of each panel and differences between cultivars, revealed by pairwise comparison, are indicated by lower case letters in (A) and (B). The two‐dimensional PCoA plot (D) visualizes bacterial community composition based on Bray–Curtis dissimilarity matrix, including significances for visualized data points in the panel. Statistics for remaining factors are listed in Table S2. [Colour figure can be viewed at wileyonlinelibrary.com]

We analysed the impact of plant traits (‘cultivar’, ‘germination rate’, ‘resistance’) and external factors (‘seed lot’, ‘country of harvest’, ‘year of harvest’) on the seed microbiota's diversity, abundance and composition. Fig. 1 visualizes samples grouped by ‘cultivar’ and ‘seed lot’, which were evaluated as the main drivers on all measures, and global statistical results are presented in Table S2.

Differences in bacterial diversity, based on Shannon *H*′ and species richness, was only significant for the host plant‐related factors ‘cultivar’ (richness: *P* < 0.0001; *H′*: *P* = 0.00001; Fig. 1A and B) and ‘resistance’ (*H′*: *P* = 0.0003), where microbiomes of resistant cultivars were significantly less diverse. Pairwise comparison of cultivars revealed significantly higher bacterial richness in seeds of cultivar A compared to B and C1, and higher Shannon diversity in A compared to B, C1, C2, C4, E, M and V (Table S2). ‘Seed lot’, modelled as a function of the fixed effect ‘cultivar’, did not affect alpha diversity in general, but had a cultivar‐specific impact on cultivar E (richness: *P* = 0.04) and M (richness: *P* = 0.04).

The difference in bacterial abundance, estimated via qPCR, was significant for the factors ‘cultivar’ (*P* = 0.003) and ‘seed lot’ (*P* = 0.014). However, estimations of abundance strongly varied across samples, reaching from 7.7 × 10^8^ to 7.0 × 10^11^ (Fig. 1C).

In contrast to diversity and abundance, bacterial community composition was significantly affected by all plant‐related and external factors tested, except for ‘year of harvest’. Based on Bray–Curtis distance, ‘cultivar’ explained 25.3% (*P* = 0.001) of variations in bacterial composition, followed by ‘seed lot’ (24.4%, *P* = 0.001) (Fig. 1D). The effect of ‘seed lot’ accounted for 31% to 50% (*P* ≤ 0.006) of the variations within the different cultivars. The factors ‘country of harvest’, ‘germination rate’ and ‘resistance’ explained each < 10% of dissimilarities between samples; however, the effects were significant (*P* ≤ 0.004).

### Taxonomic variations and core microbiota of oilseed rape seeds

Comparing higher level taxonomy of seed endophytes revealed similar profiles within samples, however, with distinct relative abundances (Fig. S1). For example, *Enterobacteriales*, which were predominant in cultivars B, C1, C2 and C4, accounted for only 9.2% of the bacterial community in cultivar V. *Betaproteobacteriales* was present in the second highest mean abundance across all samples, dominating the microbiota of cultivars A, D, M and V, but made up only 1.1% in cultivar C1. Cultivars C3 and E featured the highest abundances of *Pseudomonadales* and *Rhizobiales*, respectively. On genus level, *Serratia* revealed the highest mean abundance across all cultivars, which was mainly due to its prevalence (90% rel. abundance) in C1, whereas no ASV was assigned to *Serratia* in cultivars D and E. Overall, the microbiota of the 10 cultivars were dominated by six different genera: *Ralstonia* (dominant in cultivars A, B, D, M and V), *Serratia* (C1), *Enterobacter* (C2), *Pseudomonas* (C3), *Panotea* (C4) and *Sphingomonas* (E) (Fig. S1b).

**Fig. 2:**
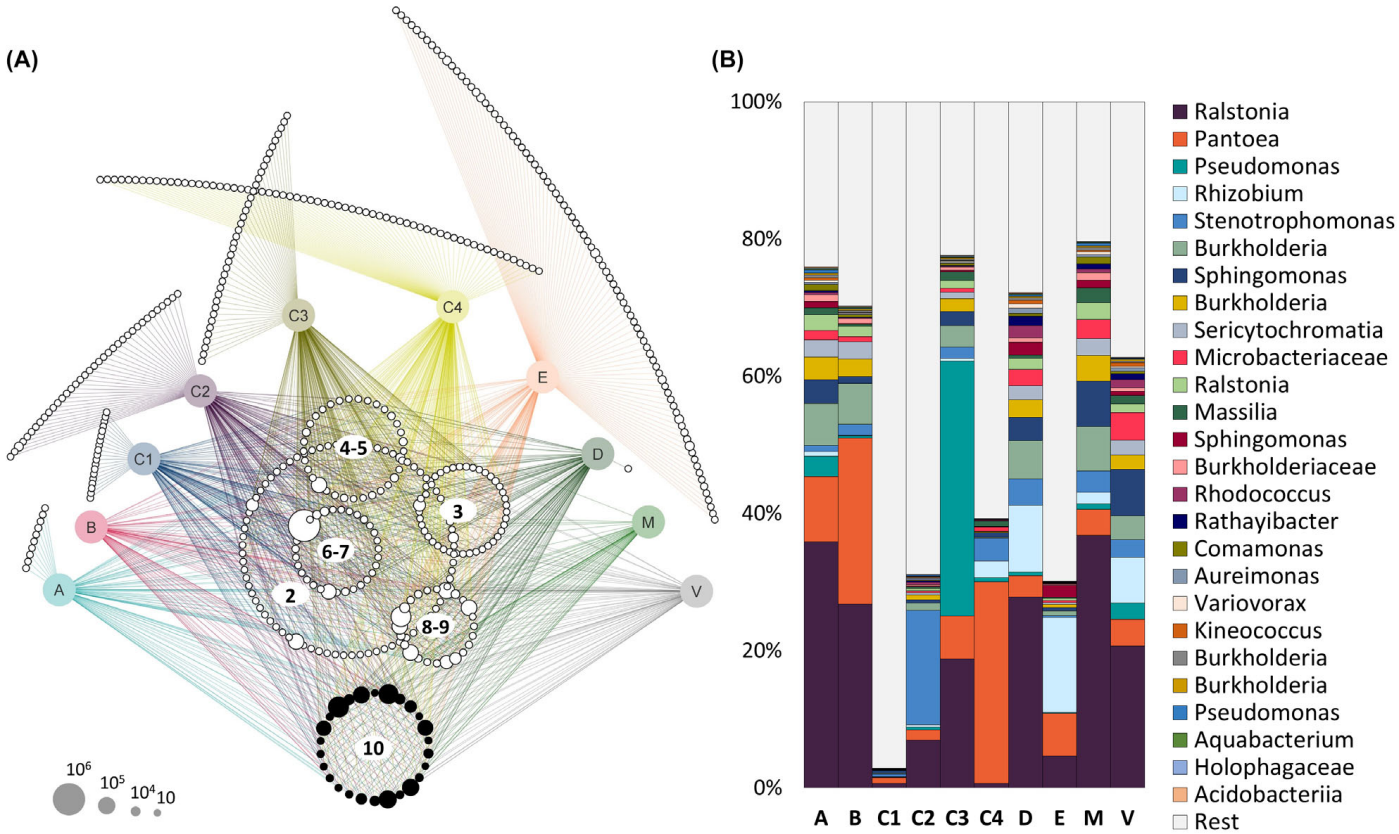
ASV profiles of oilseed rape seeds. (A) The network was constructed from the core microbiome of all cultivars (75% core) and represents unique and shared ASVs of cultivars (coloured nodes). Circles of ASVs in the network's centre highlight ASVs that were shared by two to nine cultivars and the value inside the circle indicates number of sharing cultivars. Core ASVs that were detected in all seed samples are represented as black nodes. Node size corresponds to absolute bacterial abundance in the rarefied dataset, as indicated in the legend on the lower left. Bar plot in (B) represents relative abundance of the 26 core ASVs within seeds of each cultivar; grey bars (‘Rest’) denote the fraction of ASVs that was not assigned to the core. [Colour figure can be viewed at wileyonlinelibrary.com]

To identify ASVs that were shared between all samples, *that is* the oilseed rape seed core microbiome, as well as ASVs unique for each cultivar, a network was constructed based on the combined table of each cultivars' core microbiome (75% core) (Fig. 2A). In total, 26 ASVs were assigned to the core, representing 0.1% of ASVs assigned to the overall dataset. The network analysis also highlights the partially large fraction of ASVs uniquely assigned to one specific cultivar: 12%, 13%, 20%, 17%, 26% and 49% of the assigned seed microbiota constituents were unique for cultivars A, C1, C2, C3, C4 and E, respectively. Cultivar D contained only one unique ASV and cultivars B, M and V shared all their ASVs with at least one of the other cultivars. However, the relative abundance of those unique ASVs was generally low, in contrast to core ASVs, which, despite being few in numbers, accounted for high relative abundances in most seeds (Fig. 2B). More than 60% of the microbiota in cultivars A, B, C3, D, M and V, and between 30–40% in C2, C4 and E, were represented by core ASVs. Only for C1 the fraction was low (3%). In general, ASVs assigned to *Ralstonia*, *Pantoea*, *Pseudomonas*, *Rhizobium*, *Stenotrophomonas*, *and Burkholderia* were most abundant among the core ASVs.

### Linking the host's functional characteristics to the seed microbiota

**Fig. 3:**
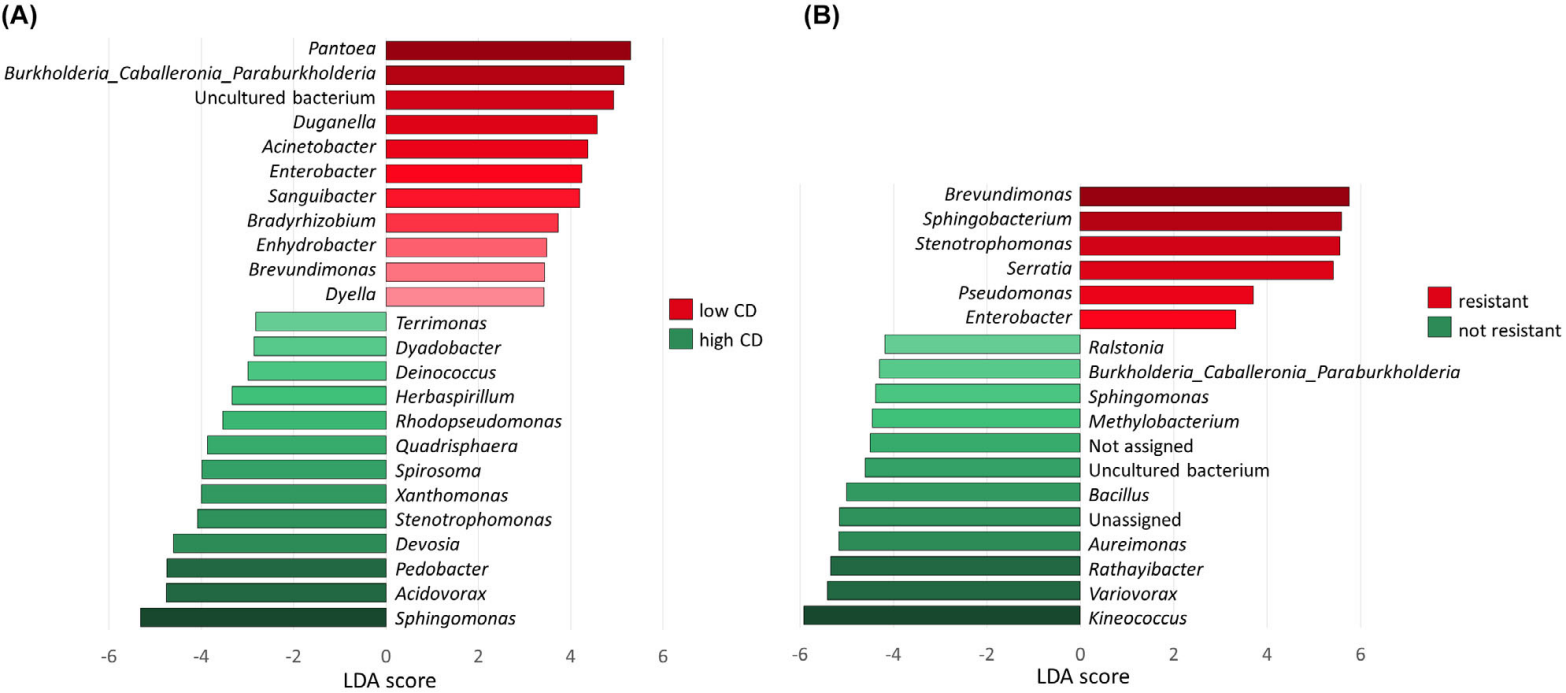
Potential bacterial marker taxa defined by LDA combined with LEfSe. Plots show bacterial genera that are significantly overexpressed (red) and under‐expressed (green) in (A) seed samples with low compared to high germination rates (CD levels) and (B) seed samples of cultivars resistant against *P. brassicae* compared to non‐resistant cultivars. Only genera with LDA scores > 2.0 and *P* < 0.05 are shown. [Colour figure can be viewed at wileyonlinelibrary.com]

Based on beta diversity estimations, the host‐related functional factors ‘germination rate’, and ‘resistance’, significantly explained variations in the bacterial composition of seeds. We performed LEfSe to analyse whether specific microbiome compositions enable discrimination between (i) high (CD rate ≥ 85%; *n* = 39) and low (CD rate < 85%; *n* = 39) germination rates, and between (ii) seeds of *P. brassicae*‐resistant (*n* = 12) and non‐resistant (*n* = 60) samples. After filtering features with at least 100 counts in all samples and a minimum prevalence of 10% in samples, 145 features remained to be tested. Applying LEfSe on ‘germination rate’ as discriminating parameter revealed 87 features, collated to 24 genera, that showed significant (*P*‐value < 0.05) correlation to either high or low CD levels (Fig. 3A). Using ‘resistance’ as testing parameter identified 18 genera (30 features) that most likely determine the differences between *P. brassicae*‐resistant and non‐resistant cultivars (Fig. 3B).

### Linking host phylogeny to the seed microbiota and estimations for microbial inheritance

**Fig. 4:**
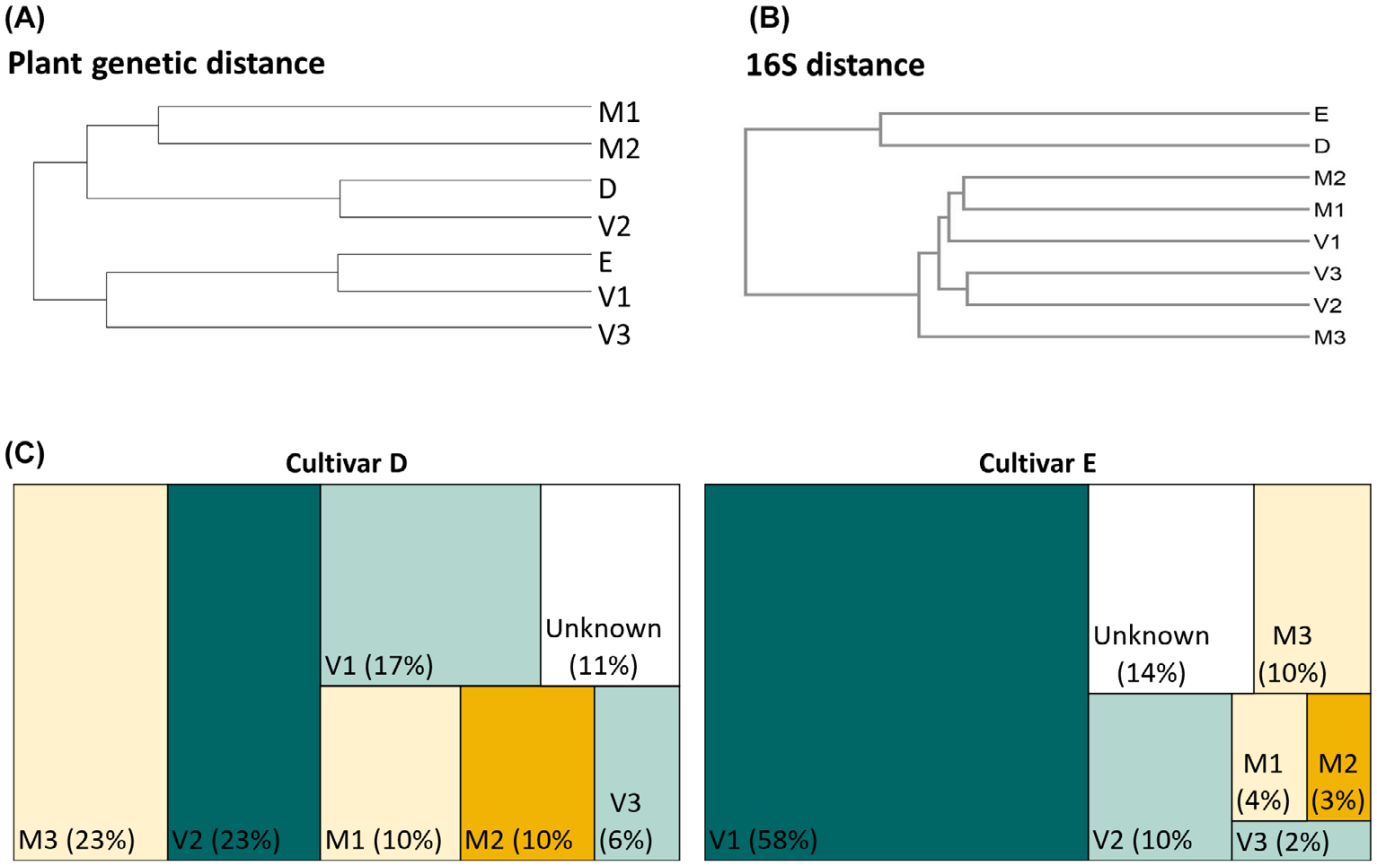
Phylogenetic and 16S rRNA gene distance and estimation of microbiota's origins. Phylogenetic tree in (A) is based on *Brassica* marker genes. (B) shows hierarchical clustering based on Bray–Curtis dissimilarity of seed microbiomes of parental lines (V, M) and hybrids (D, E). Tree map charts in (C) represents the estimated sources of potentially inherited bacteria in seeds. Bayesian approach implemented in SourceTracker2 was applied by setting all samples of cultivars M and V as sources and the hybrid samples D and E as sink. Squares of the actual maternal and paternal lines of each respective hybrid are coloured dark green and dark yellow, respectively. White squares (‘Unknown’) indicate percentage of taxa with low probability to have been transmitted from any of the sources. [Colour figure can be viewed at wileyonlinelibrary.com]

We confronted the phylogenetic distance of parental lines and hybrids of oilseed rape cultivars to Bray–Curtis distance of bacteria associated with seeds of those cultivars (Fig. 4A and B). Host phylogenetic distance, based on Modified Rogers Distance of genetic *Brassica* markers, revealed hybrids that share the same maternal line to cluster close to their paternal lines. Bacterial community clustering, in contrast, resulted in one cluster for hybrids and one cluster for parental lines, indicating that both parents contribute to the seed microbiota of hybrids, which consequently are resembling each other.

A community‐wide Bayesian model (SourceTracker 2) was applied to estimate the fraction of potentially inherited seed microbiota, by including both, the actual parental seed lots (D = M2 × V2; E = M2 × V1) and those seed lots from cultivars M and V, which were not used to produce hybrid seeds. The models estimated that both cultivars D and E obtained a large percentage of ASVs from their actual paternal line (23% and 58%, respectively) (Fig. 4C). Since all M and V seed lots represent the same cultivar, adding up the estimated values of microbial sources is reasonable and results in high percentages of ASVs that have been transmitted to the hybrids (D: 89%, E: 86%), compared to the percentage of ASVs that unlikely originated from these parental lineages (D: 11%, E: 14%).

## Discussion

**Fig. 5:**
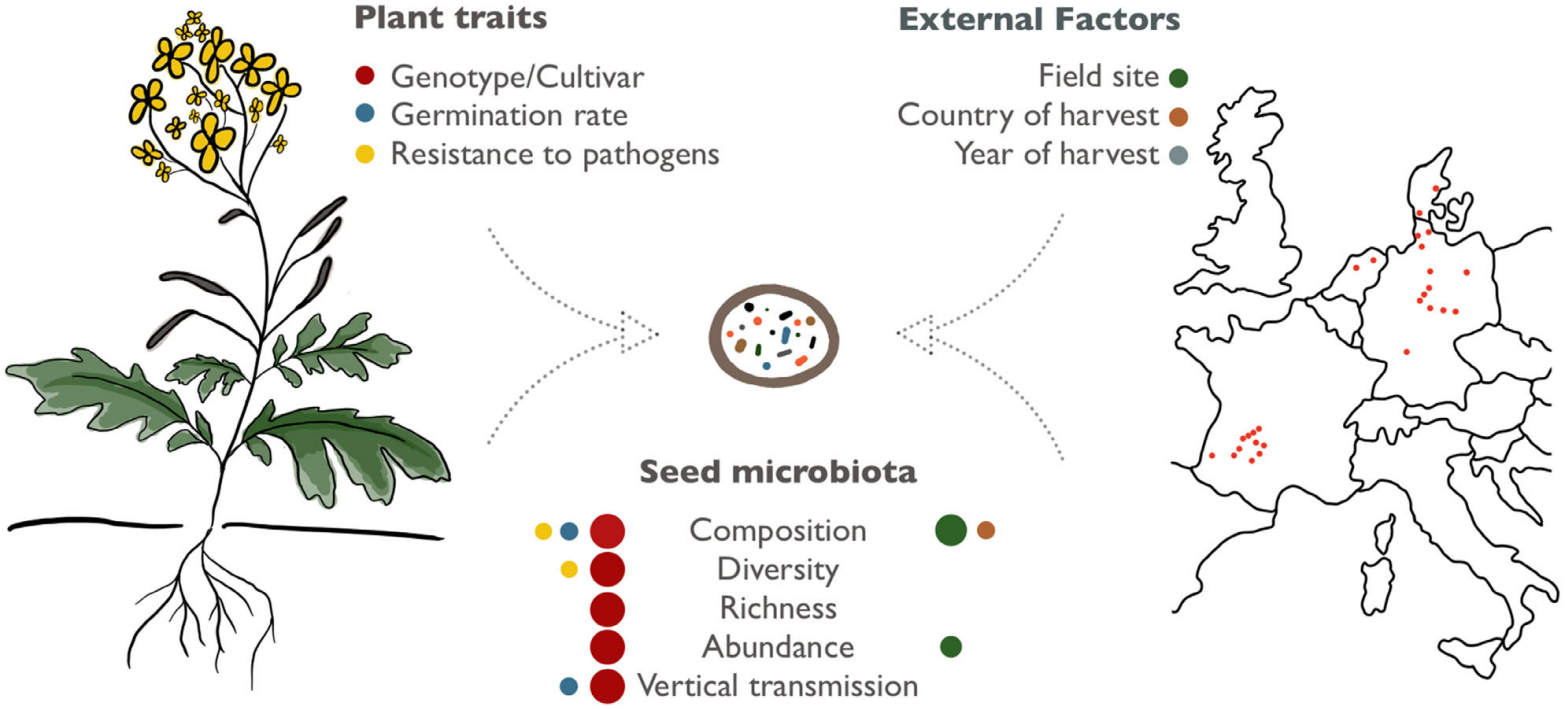
Illustration of the study's main findings. Plant traits (left) and external factors (right) were analysed to contribute to the structure of the *B. napus* seed microbiome. Significant impacts of colour‐coded factors on endophyte composition, diversity, richness, abundance and vertical transmission are indicated by coloured dots, where greater size of dots indicates higher relevance of the respective factor for seed microbiota characteristics. The map on the right indicates the 26 European field sites sampled for the present study. [Colour figure can be viewed at wileyonlinelibrary.com]

In this study, we disentangled the impact of plant traits (genotype, germination rate, resistance to pathogens) and external factors (field site, country of harvest, year of harvest) on the structure of the *B. napus* seed microbiome. The host genotype was determined as the main factor explaining the variations in bacterial composition, diversity, richness and abundance. Besides the cultivar, only the host's resistance against *P. brassicae* affected bacterial diversity. In contrast, endophyte composition correlated to all factors tested, except the year of seed harvest, which had no impact on the seed microbiota on any measure (Fig. 5). While significant impacts of cultivar, field site and soil and environmental conditions on the oilseed rape seed microbiota have been reported previously (Rybakova *et al*., 2017; Rochefort *et al*., 2019; Morales Moreira *et al*., 2021), our study revealed for the first time that the cultivar is a main driver of bacterial diversity, and that host's functional characteristics (resistance towards *P. brassicae* and germination rates) correlate to seed endophyte composition, where bacterial biomarkers were identified for both. Moreover, we estimated the contribution of parental breeding lines to seed microbiota of their hybrid offspring and found that the paternal parent might play an important role during the assembly of the seed microbiota, with potential impact on the offspring's germination performance.

Our findings on the primary importance of host genotype, followed by a significant, but subordinate impact of environmental factors, are in contrast to previous studies on oilseed rape seed microbiomes. For example, a recent study that combined oilseed rape seed endo‐ and epiphytes, described the environment to be the main driver, followed by significant impact the cultivar (Rochefort *et al*., 2019). Another study reported that seed epiphytes were only driven by environmental factors, without any influence of the cultivar (Morales Moreira *et al*., 2021). In conjunction with our data, a gradient for environmental relevance, declining from outer to inner seed tissues, might be proposed, where host genetics mainly determine the very specific microbiota that is vertically transmitted to internal seed tissues (Newcombe *et al*., 2018). This is a relevant finding for microbiome‐assisted breeding; here, seed endophytes should be rather considered than epiphytes. Despite the large compositional differences between cultivars, a small (0.1% of total ASVs) but abundant core microbiota was identified and assigned to taxa such as *Pantoea*, *Pseudomonas*, *Rhizobium*, *Stenotrophomonas*, *and Burkholderia*. Since those taxa are being reported frequently to colonize seeds, we support the idea of a symbiotic relationship and their relevance for plant health in general (Hardoim, 2019; Batista and Singh, 2021). The impact of the field site was significant for bacterial composition and abundance. Certainly, the microbiota transmitted from parental lines to seeds are also dependent on the microbial pool available in the environment. In our dataset, the field site explained between 31% and 50% of the variations within cultivars. However, based on our current dataset, we are unable to determine the impact of a certain field site, since the 27 seed lots were harvested from 26 fields, accordingly, only one field was sampled twice for seed lots of cultivar E and M. Yet, their associated microbiota did not resemble each other and clustered distinct in ordination plots and dendrograms. The country of harvest revealed a less important, but significant impact on bacterial composition as well. This might be explained by the close proximity of some field sites, especially in France and Germany, where regional parameters, such as temperature and precipitation regimes, likely affect the microbiota present.

Among all plant‐related and external factors tested, only the year of seed harvest did not correlate to seed endophyte composition or diversity. This was surprising and underlines the minor impact of environmental factors in our study. In addition, we did not observe the expected decline of seed‐associated microbial diversity after storage (Shade *et al*., 2017). It should be noted in this context that our study does not discriminate between dead and viable bacteria.

We additionally demonstrated a correlation of host functional characteristics and seed endophytes. Both, germination rates and resistance against *P. brassicae*, showed significant associations with the seed microbiota composition. Bacterial biomarkers for both factors were identified, and a functional potential for host health might be inferred for some of them. Especially *Sphingomonas* and *Stenotrophomonas*, both biomarkers for high germination rates, are often considered as beneficial for the host plant, whereas *Ralstonia* and *Burkholderia*, both biomarkers for low germination rates, are rather known for adverse impact on plants (Hardoim *et al*., 2015; Compant *et al*., 2019; Saikkonen *et al*., 2020). The plant's resistance to *P. brassicae* might also select for functionally adapted members of the microbiota, such as *Sphingobacterium*, *Stenotrophomonas*, *and Pseudomonas*; all of which, but especially the latter, represent important biofungicides (Berg *et al*., 2021). Moreover, the corresponding bacterial families (*Sphingobacteriaceae*, *Xanthomonadaceae*, *Pseudomonadaceae*) were found to be significantly more abundant in asymptomatic compared to symptomatic roots of *B. napus* plants that were infected by *P. brassicae* (Zhao *et al*., 2017). Thus, a plant's resistance to pathogens may not entirely be attributed to host genetics, which has also been demonstrated in the two following studies. Matsumoto *et al*. (2021) proved that pathogen resistance of rice plants is conferred by a seed‐endophytic, trans‐generationally inherited *Sphingomonas* strain and Mendes *et al*. (2018) observed that pathogen resistance of common bean correlates to higher abundance of antagonistic bacteria in the rhizosphere. The authors proposed that breeding for resistance may have unintentionally altered the microbiome, which acts as primary defence line to protect against pathogen colonization. Also, in our dataset, *P. brassicae* resistance is based on plant traits; however, resilience might be reinforced by the microbial component of the holobiont. Since seed microbiota likely take priority over environmental microbiota to colonize the emerging seedling (Abdelfattah *et al*., 2021b), their defence force might even exceed that of the rhizosphere microbiota.

Our data furthermore suggest that certain functional characteristics of the holobiont, might be even transferred to the offspring via microbial hybridization; an idea that has already been suggested for other plants (Liu *et al*., 2013; Adam *et al*., 2018; Kusstatscher *et al*., 2021; Abdelfattah *et al*., 2021a). We applied a Bayesian approach on two hybrids (E, D), which were produced from the same maternal line (M2) but from different paternal lines (V1, V2), to identify the fraction of endophytes that potentially originated from either parent. The contribution of the paternal line was generally higher (24%–74%) than the contribution of the maternal parent (3%–10%). Interestingly, those estimations correlate also to functional similarities between paternal lines and their hybrids: V1 and its hybrid E revealed high germination rates, whereas V2 and its hybrid D performed weakly. Moreover, the hybrids´ performance was irrespective of the field location; each of them was produced on four different field sites. While for plant genetics in general, the inheritance mode of traits and the transmission of parental alleles to hybrids is still a matter of debate, a study on *Arabidopsis thaliana* showed that the contribution of maternal and paternal genomes during early plant development stages is non‐equivalent and that the impact of paternal gene activity is unexpectedly profound (Del Toro‐De León *et al*., 2014). Our observation of such strong importance of the paternal parent for seed microbiome assembly is novel, has application potential and requires further research.

## Conclusion

In conclusion, we demonstrated that the cultivar is a main driver of bacterial diversity in *B. napus* seeds, and that hybridization of the maternal and especially the paternal microbiota might play an important role during the assembly, with potential impact on the offspring's health. We identified microbial biomarkers for germination performance and resistance towards plant pathogens, which are not yet available and can be used for combined biocontrol and microbiome management strategies. Thereby, our results contribute to plant microbiome research in general and have substantial implications for microbiome‐assisted breeding approaches for oilseed rape.

## Experimental procedures

### Seed origin and metadata

**Fig. 6:**
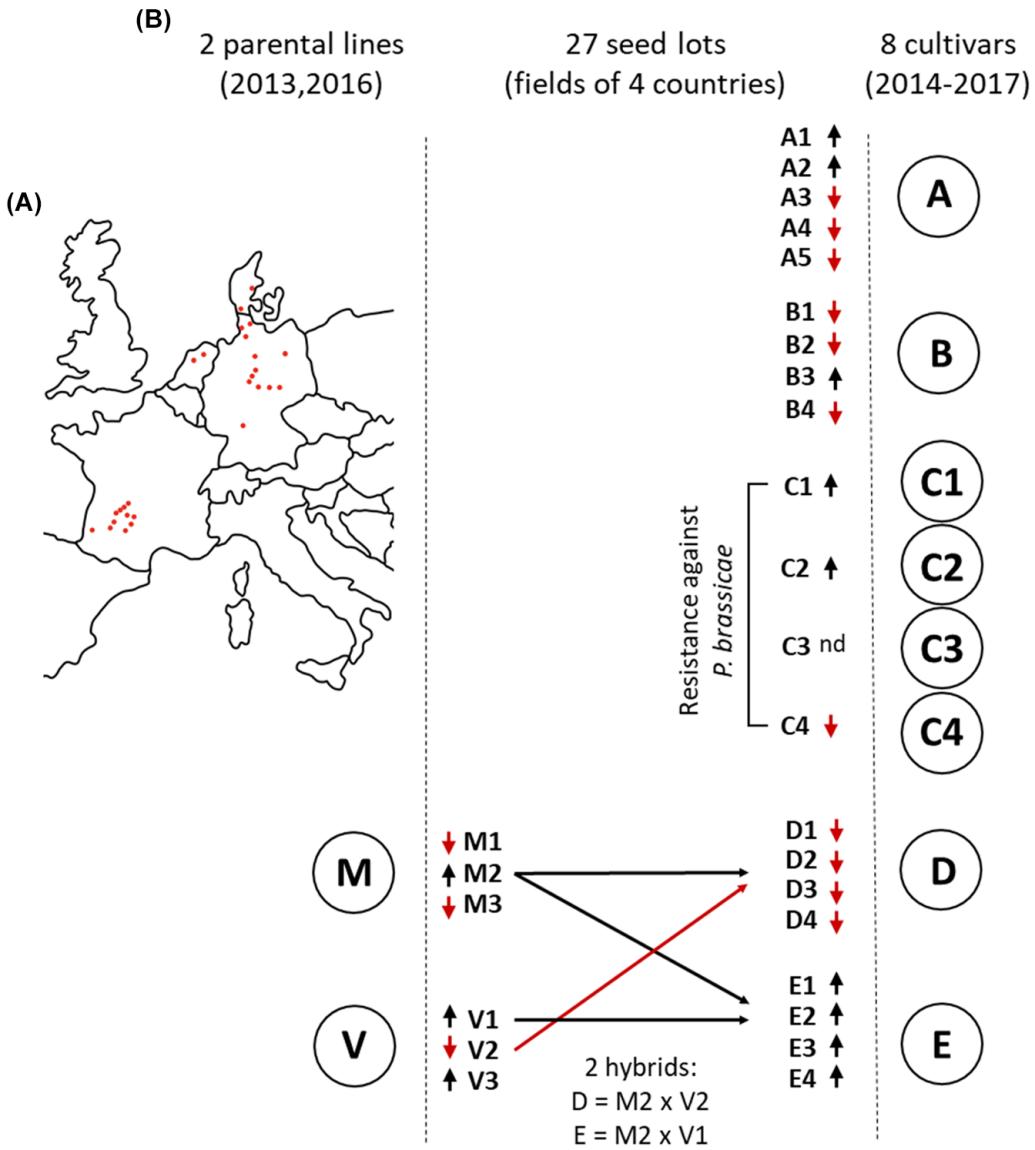
Overview of the oilseed rape seed samples investigated. A. Illustration of the 26 field sites in four European countries. B. General metadata of cultivars A, B, C1, C2, C3, C4, D, E, M and V. Cultivars are represented by one to five seed lots, where each seed lot represents seeds of one cultivar sampled from a different field. Black arrows pointing up and red arrows pointing down, next to seed lot IDs, denote for high (≥ 85%) and low (< 85%) germination rates, evaluated in CD vigour tests (nd = not detected). C1 to C4 are to be differentiated from remaining cultivars by an inbred resistance against *P. brassicae*. Arrows pointing from M2 (maternal line) and from V1 and V2 (paternal lines) to D and E (hybrids) seed lots indicate parental lines and their half‐sib hybrid offspring. [Colour figure can be viewed at wileyonlinelibrary.com]

Seeds of 10 oilseed rape cultivars (A, B, C1, C2, C3, C4, D, E, M, V) harvested between 2013 and 2017, from 26 fields in four European countries, were obtained from NPZ (Norddeutsche Pflanzenzucht Hans‐Georg Lembke KG). General information and sample origins are outlined in Fig. 6, while detailed metadata are provided in Table S1. Each cultivar was obtained from one to a maximum of five different seed lots, where a seed lot denotes for seeds of one cultivar, sampled from one field. Seeds for each seed lot were sampled in three biological replicates. We investigated the impact of ‘cultivar’, ‘seed lot’, ‘country of harvest’, ‘year of harvest’, ‘resistance’ and ‘germination rate’ on seed endophytes. ‘Resistance’ refers to inbred resistance against *Plasmodiophora brassicae*, which differentiates the cultivars C1, C2, C3 and C4, from the remaining cultivars. ‘Germination rate’ was evaluated under standardized controlled deterioration (CD) vigour test protocol according to the ISTA Rules, Chapter 15.8.3. CD germination rates, ranging from 13% to 96% in seed lots, were grouped into ‘high CD’ (CD rate ≥ 85%) and ‘low CD’ (CD rate < 85%). Specific seed lots of the parental lines M and V, harvested in 2013, were planted in 2017 on eight different fields to produce the hybrids D and E. In detail, cultivar D (low CD) is a hybrid of M2 (high CD) and V2 (low CD), and cultivar E (high CD) is a hybrid of M2 (high CD) and V1 (high CD); hence, both hybrids are half‐siblings sharing the same maternal line, but having different paternal lines. The fraction of the vertically transmitted microbiome from each parent was assessed for those samples.

### DNA extraction and library generation

The total DNA was extracted from all seed samples. For each sample, 20 seeds per replicate were surface sterilized by incubating them in sodium hypochloride (2%) for 5 min under agitation, following by six washing steps in sterile water. Seeds were mortared under sterile conditions and the homogenized solution was pelleted and further used for DNA extraction. Utilizing the FastDNA SPIN Kit for Soil and the FastPrep Instrument (MP Biomedicals, Santa Ana, CA, USA), the total DNA was extracted following the manufacturer's protocol. DNA was quality checked using a Nanodrop 2000 (Thermo Scientific, Wilmington, DE, USA) and stored at −20°C for PCR reactions.

Isolated DNA was used for amplification of the 16S rRNA gene V4 hypervariable region with the 515f/806r primer pair (515f: 5′‐GTGYCAGCMGCCGCGGTAA‐3′; 806r: 5′‐GGACTACNVGGGTWTCTAAT‐3′) (Caporaso *et al*., 2011; Parada *et al*., 2016). All PCR reactions were performed in triplicates. The PCR mix was amplified in 35 cycles at 94°C denaturation for 45 s, 50°C annealing for 60 s and 72°C elongation for 90 s. Additionally, peptide nucleic acid PCR clamps (PNA) were used to block the amplification of plastid and mitochondrial 16S rRNA gene sequences of plants during the PCR amplification. The amplicons were purified using the Wizard SV Gel and PCR Clean‐Up System (Promega, Madison, WI) and pooled in equimolar concentrations. The paired‐end Illumina MiSeq sequencing of the barcoded Illumina library was performed by GATC Biotech (Berlin, Germany).

### Bioinformatic pipeline

Paired‐end reads were quality checked and demultiplexed using cutadapt (Martin, 2011). Bioinformatic analysis for amplicon sequencing analysis was performed using the open‐source Qiime2 version 2018.4.0 pipeline (https://qiime2.org). Primer sequences were removed. Using the DADA2 algorithm in QIIME2 the reads were quality filtered and denoised, forward and reversed reads were joined, chimera were filtered, and feature table and representative sequences (amplicon sequences variants (ASVs)) were generated (Callahan *et al*., 2016). The generated ASVs were classified using the vsearch algorithm and the SILVA v132 database as a reference (Quast *et al*., 2012; Rognes *et al*., 2016). All taxonomy‐related results (bar charts, OTU networks and results generated by LEfSe (linear discriminant analysis effect size)) were produced from the merged core microbiome of each cultivar, which was generated by keeping ASVs that were present in at least 75% of a cultivar's replicates. A network showing unique, shared and core bacterial ASVs of oilseed rape cultivars was constructed using Cytoscape v3.8.2 (Shannon, 2003).

### Statistical analyses

To calculate bacterial diversity, based on number of observed species and Shannon *H*′ index, the dataset was rarefied to 10 000 reads per sample using the rarefy_even_depth function of R package Phyloseq v.1.32.0. MetagenomeSeq's cumulative sum scaling (CSS) (Paulson *et al*., 2013) was applied to correct uneven sequencing depth for beta diversity calculations, including Bray–Curtis dissimilarity, permutational multivariate analysis (PERMANOVA) and hierarchical clustering analysis. Each ‘seed lot’ represents seeds of one cultivar from a unique field, except one field in Germany from which two cultivars were sampled. Additionally, five of the cultivars were sampled from only one country. Thus, to evaluate the impact of the factors ‘seed lot’ and ‘country of harvest’ on the seed microbiome richness, diversity, abundance and composition, we modelled those variables as a function of the fixed effect ‘cultivar’, using the function *aov* in R Package stats v.4.0.1 in R v.3.6.2 (R Core Team, 2020). Means of factor groups were compared using Tukey multiple comparisons. Differences in the microbiome communities were tested using the function *adonis2* (PERMANOVA) in the package VEGAN (Oksanen *et al*., 2007). Pairwise comparisons were done using R package PAIRWISEADONIS (Arbizu, 2019). Differences between seed lots within one cultivar were tested by applying *adonis* test on subsets for each cultivar.

Bacterial genera correlating significantly to host functional parameters ‘germination rate’ and ‘resistance’ were identified by LEfSe as implemented in MicrobiomeAnalyst (Segata *et al*., 2011; Kassambara and Mundt, 2017; Chong *et al*., 2020). Only features with a minimum count of 100 and a prevalence in 10% of the samples were included, and a threshold of 2 and a *P*‐value of 0.05 were set for the linear discriminant analysis (LDA).

Hierarchical clustering of the seed microbiome is based on Bray–Curtis dissimilarity. We used *hclust* in R package Stats v.4.0.1 with ‘average’ as clustering method, and visualized results with *fviz_dend* function in R package Factoextra v.1.0.7 (Kassambara and Mundt, 2017). The phylogenetic tree representing genetic relatedness of cultivars is based on calculating Modified Rogers Distance using marker information from the Illumina Infinium 15 K‐Chip (*Brassica*).

To evaluate the potential of the seed microbiota to be inherited from parental lines, Sourcetracker2 (Knights *et al*., 2011) was applied on a subset of the data. The subset contained all samples of parental lines M and V, including the actual seed lots M2, V1 and V2, as well as seed lots which were not used to produce hybrid seeds (M1, M3, V3). M and V samples were set as sources and the hybrid samples D1, D2, E3 and E4 were set as sink.

### Quantitative real‐time PCR (qPCR)

To quantify bacteria in seed samples, a qPCR approach based on fluorescence detection was performed in a Rotor‐Gene 6000 real‐time rotary analyser (Corbett Research, Sydney, NSW, Australia). Each sample was analysed in independent triplicates and reaction mixtures contained 5 μl KAPA SYBR Green, 0.5 μl (10 μM each) of each primer (515f–927r (Köberl *et al*., 2011)), 1 μl template DNA, adjusted with PCR‐grade water to a final volume of 10 μl. The following temperature settings were used: 95°C for 3 min, 30 cycles of 95°C for 5 s, 54°C for 20 s, 72°C for 5 s and a final melt curve of 72–96°C. Gene copy numbers that were detected in negative control samples were subtracted from the respective run.

## Conflict of interest

None declared.

## Author contributions

BW analysed data and wrote the manuscript. AAbdelfattah and HM contributed to data analysis and interpretation of results. PK and WW wrote a first draft of the manuscript and TC discussed the results. SG, AAbbadi and SR designed the sampling and performed germination assays. GB designed the study, contributed to the interpretation of results and wrote the manuscript.

## Data availability

These sequence data have been submitted to the European Nucleotide Archive (ENA) under the project number PRJEB51924.

## Supporting information


**Fig. S1.** Taxonomic composition of oilseed rape seed endophytes. Barcharts in (a) and (b) represent the 18 most abundant bacterial families, and the 25 most abundant genera across the dataset, respectively; remaining genera are summarized as others.
**Table S1.** Detailed metadata for the different seed lots.
**Table S2.** Diversity and abundance dissimilarities between seeds for host‐related and external factors.
