# Supplementary material for: The Brassica napus seed microbiota is cultivar‐specific and transmitted via paternal breeding lines
Source: Microb Biotechnol. 2022 May 20;15(9):2379–90. doi: 10.1111/1751-7915.14077 (PMC9437892; doi:10.1111/1751-7915.14077)
Supplement: Supplementary file 1 — Fig. S1. Taxonomic composition of oilseed rape seed endophytes. Barcharts in (a) and (b) represent the 18 most abundant bacterial families, and the 25 most abundant genera across the dataset, respectively; remaining genera are summarized as others. Table S1. Detailed metadata for the different seed lots. Table S2. Diversity and abundance dissimilarities between seeds for host‐related and external factors. [file MBT2-15-2379-s001.docx]

**Supplementary Material for the article**

**The Brassica napus seed microbiota is cultivar-specific and transmitted via paternal breeding lines**

Birgit Wassermann^ab*^, Ahmed Abdelfattah^c^, Wisnu Adi Wicaksono^b^, Peter Kusstatscher^b^, Henry Müller^c^, Tomislav Cernava^b^, Simon Goertz^d^, Amine Abbadi^d^, Steffen Rietz^d^, Gabriele Berg^bce^

**Table S1** Detailed metadata for the different seed lots.


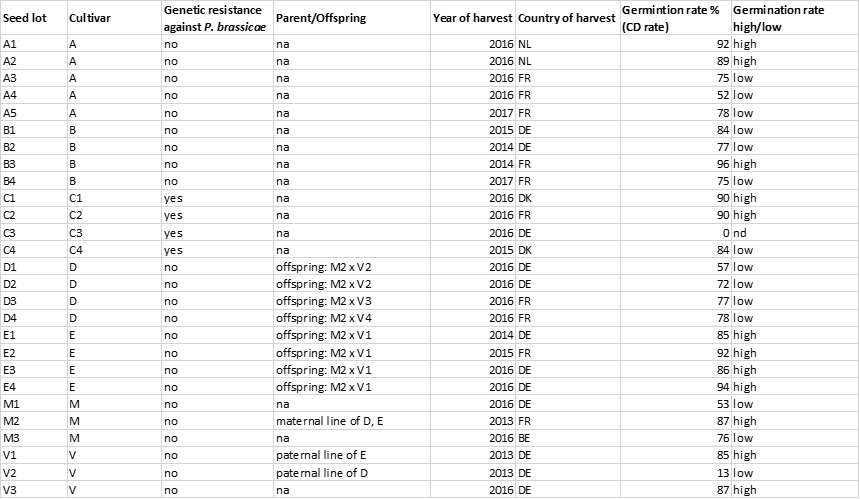


na = not applicable, nd = not detected

**Table S2** Diversity and abundance dissimilarities between seeds for host-related and external factors.

*The responsive variables ’seed lot’ and ‘country of harvest’ were modeled as a function of the fixed effect ‘cultivar’. Cultivar-specific impacts of factors were not calculated for C1, C2, C3, and C4 as they were each represented by only one seed lot. No statistics were calculated when values for the factors tested were equal within a cultivar, indicated as ‘na’ (=not applicable). Significances are highlighted in bold.

**Fig. S1** Taxonomic composition of oilseed rape seed endophytes. Barcharts in **(a)** and **(b)** represent the 18 most abundant bacterial families, and the 25 most abundant genera across the dataset, respectively; remaining genera are summarized as others.

**
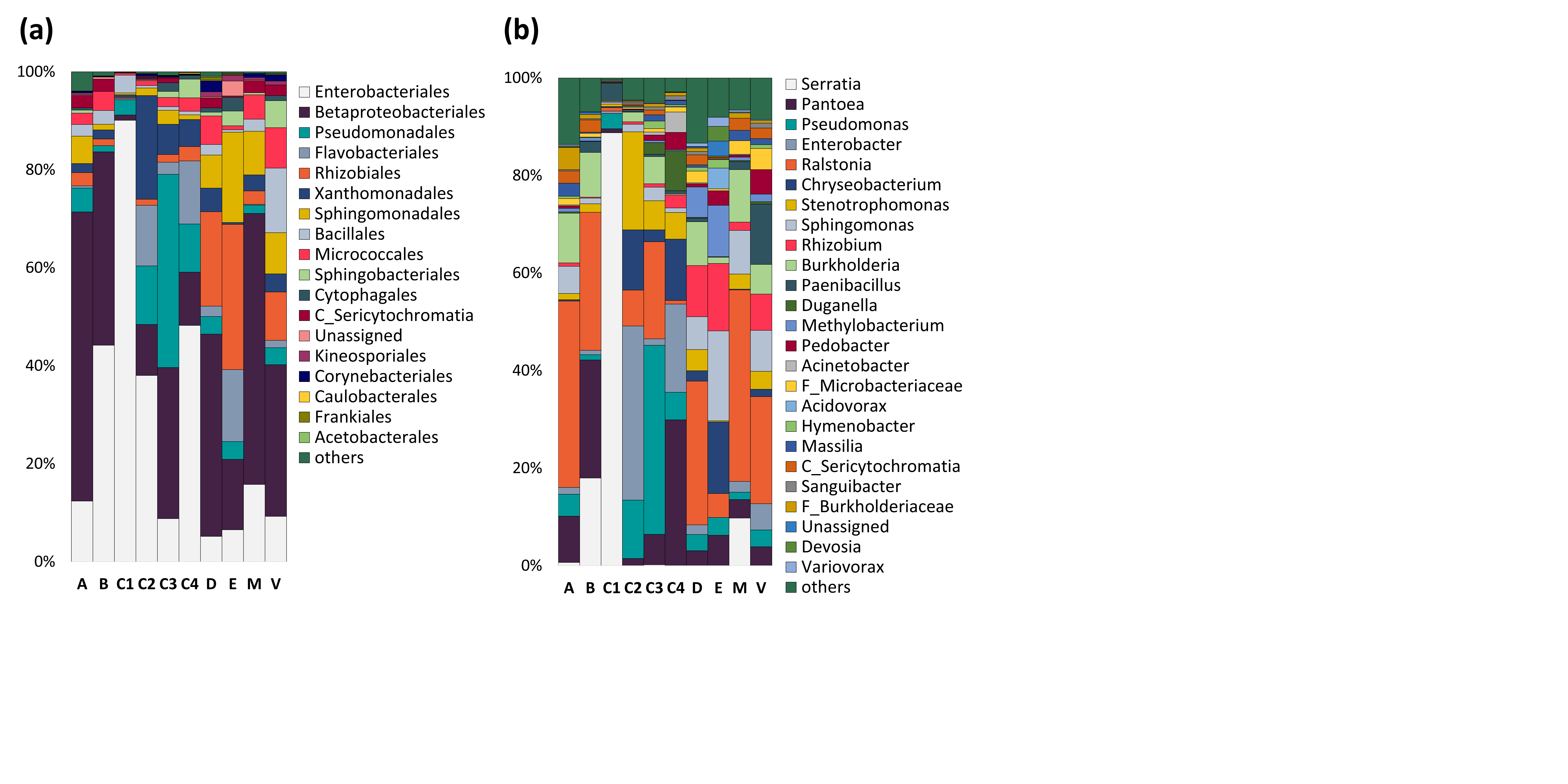
**
